# Novel Denisovan and Neanderthal Retroviruses

**DOI:** 10.1128/JVI.01825-14

**Published:** 2014-11

**Authors:** Adam Lee, Derek Huntley, Pakorn Aiewsakun, Ravinder K. Kanda, Claire Lynn, Michael Tristem

**Affiliations:** a Imperial College London, South Kensington Campus, London, United Kingdom; b Imperial College London, Silwood Park Campus, Ascot, Berkshire, United Kingdom

## Abstract

Following the recent availability of high-coverage genomes for Denisovan and Neanderthal hominids, we conducted a screen for endogenized retroviruses, identifying six novel, previously unreported HERV-K(HML2) elements (HERV-K is human endogenous retrovirus K). These elements are absent from the human genome (hg38) and appear to be unique to archaic hominids. These findings provide further evidence supporting the recent activity of the HERV-K(HML2) group, which has been implicated in human disease. They will also provide insights into the evolution of archaic hominids.

## TEXT

In 2008, an archaeological dig at a cave in the Siberian Altai mountain range led to the discovery of a finger bone belonging to a female hominid, dating to at least ∼50,000 years ago (1, 2). From this, the DNA of a subspecies of Homo sapiens, designated Denisovans, was sequenced (1). Similarly, a draft Neanderthal genome—a sister group to Denisovans—–was sequenced from three individuals in 2010 (3, 4). Using this data, Agoni et al. (5) identified 14 novel human endogenous retrovirus K (HERV-K) proviruses, which were absent from the human genome sequence (assembly hg19). The authors suggested that these HERVs were unique to archaic hominids and that no orthologous insertions would be found in modern humans (5). Subsequently, however, Marchi et al. reported that all of these sequences were actually present, or likely to be present, in some modern humans (6).

In this study, we screened the most recently available high-coverage genomes for a Denisovan (4) and an Altai Neanderthal (2) for HERV-K proviruses. We present six novel, endogenized retroviruses, absent from the hg38 human genome, 43 modern-human genomes reported by Lee et al. (7), and a further 358 reported by Marchi et al. (8). These may therefore represent the first proviruses unique to Neanderthal and Denisovan hominids.

While endogenized retroviral DNA makes up ∼8% of the human genome, only one group—HERV-K(HML2)—appears to have been active within the past million years. This has been demonstrated by the observation that some members of this group, but not others, are insertionally polymorphic, having been identified in some modern humans (9). Although no active, replication-competent HERV-K(HML2) elements have been identified to date, it remains possible that such elements exist and may cause disease in some modern humans.

The high-coverage Neanderthal and Denisovan genomes screened in this study were both derived from fossils found in Denisova Cave (2, 4). These genome sequences consist of short, unassembled DNA reads averaging ∼70 to 200 bp and were sequenced to 52- and 30-fold coverage, respectively (versus 1.3-fold [3] and 1.9-fold [1] coverage for the genomes screened previously by Agoni et al. [5]). They therefore likely represent almost-complete genome coverage.

Novel retroviral insertions in archaic hominids can be recognized when orthologous flanking DNA in modern humans is not interrupted by a HERV insertion, manifesting instead as an empty preintegration site. We obtained reads containing 5′ host-virus junctions using a perl script that stringently detected the first 20 bp of the start of the HERV-K(HML2) long terminal repeat (LTR), allowing us to build libraries of reads containing hominid and viral DNA. Flanking sequences were then extracted and BLAST searched against the human genome (hg38), using blastn and a word size of 11. Apparent novel HERV-K(HML2) insertions were identified by a lack of retroviral sequence downstream of a matching flank in the modern-human genome.

To confirm that putative novel insertions were not a result of sequencing artifacts, such as template switching, we used three approaches. First, we confirmed that each retrovirus was represented by multiple reads, as this would be unlikely to occur in the event of sequencing error. Second, we attempted to identify the corresponding 3′ flanks for each candidate provirus. This involved obtaining the modern-human sequence directly downstream of the flank-virus breakpoint and locating matching sequence in the Denisovan and Neanderthal genomes. Matching reads containing LTR sequence directly upstream of the 3′ flank were extracted using BLAST, utilizing word sizes of 5 to 7. This enabled matches to be returned, despite small mismatches occurring due to target site duplications (TSDs) at the virus-host junction. Lastly, the presence of matching TSDs was considered additional verification of the virus. Conversely, we then repeated each of these steps with the 3′ end of the HERV-K(HML2) LTR to identify 3′ host-virus junctions. Sequence reads are given in Table S1 in the supplemental material.

Using this approach, we identified a total of nine HERV-K(HML2) proviruses present within the Neanderthal and Denisovan genomes—while also absent from hg38—that were not reported by Agoni et al. (5). However, their absence from the hg38 sequence does not necessarily imply absence from all modern humans; such elements could be insertionally polymorphic, as demonstrated by Marchi et al. (6). While we did not directly screen further modern-human genomes, we compared our nine elements against those recovered by Lee et al. (7) and Marchi et al. (8) from their analyses of high-coverage modern-human genomes. This revealed that three of these viruses were present within the data of Lee et al. (7), of which two were also identified by Marchi et al. (8).

**FIG 1:**
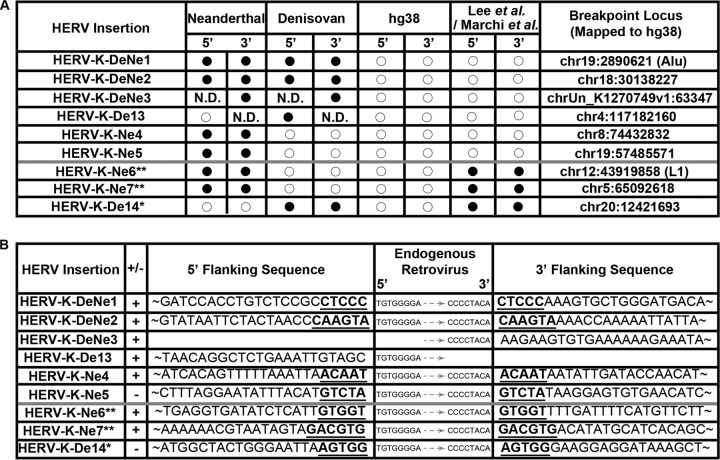
(A) Distribution of the archaic hominid insertions within Neanderthals and Denisovans, compared to hg38 and the HERV-K elements recovered in previous studies (6–8). Filled circles denote that an element is present, while open circles denote absence. N.D. indicates that there were no sequence data available. The loci of corresponding empty preintegration sites, mapped to hg38, are also given. If this occurred within a repeat, the class of repeat is listed in parentheses. Single asterisks adjacent to the last three viruses represent those elements that were also recovered in some modern humans by Lee et al. (7), while double asterisks represent those viruses also recovered by Marchi et al. (8). The distribution of these three elements is likely explained by ancestral polymorphism. (B) HERV-K(HML2) flanking sequences for the nine endogenized retroviruses identified here. The 5′ and 3′ flanking regions are shown, together with the proximal and distal ends of the HERV LTR. Nomenclature follows the convention and numbering set by Agoni et al. (5). Reads where flanking sequence was extensive were truncated and are provided in full in Table S1 in the supplemental material. + or − denotes the native orientation of the read against hg38. For proviruses for which both 5′ and 3′ flanks were obtained, the matching TSD sequence is underlined and in boldface.

The remaining six elements therefore appear to be absent from both hg38 and the additional 401 modern-human genomes investigated in previous reports (7, 8). For four of these elements, both the 5′ and 3′ virus-host junctions were identified, while the remaining two—which were both represented by multiple sequence reads—were derived from single ends. Of the six proviruses, three were shared by both Denisovans and Neanderthals, while two were unique to Neanderthals and one to Denisovans (Fig. 1). We note that one of these proviruses, De13, is located approximately 1 kb upstream of an existing HERV-K(HML2) solo LTR in hg38; it also appears to share the same TSD, as well as a similar flanking sequence. This would normally suggest that it is a sequencing artifact. However, it is represented by multiple sequence reads in the Denisovan genome and lies within a highly repetitive region. Its flanking sequence exactly matches the region 1 kb upstream of the known solo LTR in hg38.

As a result of genetic drift, neutral HERV insertions can become fixed in a population within a time frame dependent on population size and generation time. It is estimated that the average time taken to fixation in humans is ∼800,000 years (10). Since modern humans are estimated to have diverged from Denisovan and Neanderthal lineages approximately 553,000 to 589,000 years ago (2), we would expect that some—but not all—of the novel HERV-K(HML2) elements in these archaic hominids would be absent in modern humans. This is consistent with our results; six elements appear to be absent from all of the modern-human genomes investigated to date, whereas others (identified in this and previous reports [5, 7, 8]) are present within some of them. However, it remains possible that these six elements are also present in modern humans, albeit at very low allele frequencies.

We suggest that at least some of the six proviruses identified in this study inserted into archaic hominids after their divergence from modern humans; however, it is also possible that they inserted before the divergence of archaic hominids and modern humans, with these ancestral polymorphisms being subsequently lost from modern humans by genetic drift. These findings will help improve our understanding of archaic hominid evolution and provide additional insight into the recent activity of the HERV-K(HML2) retroviral group.

## Supplementary Material

Supplemental material
