# Supplementary material for: Novel Denisovan and Neanderthal Retroviruses
Source: J Virol. 2014 Nov;88(21):12907–9. doi: 10.1128/JVI.01825-14 (PMC4248912; doi:10.1128/JVI.01825-14)
Supplement: Supplemental material [file supp_88_21_12907__index.html]

Novel Denisovan and Neanderthal Retroviruses — Supplemental material 

# Novel Denisovan and Neanderthal Retroviruses

## Supplemental material

**Files in this Data Supplement:**

- Supplemental file 1 -

  Table S1 (Sequence reads of the 9 identified proviruses and read names as given in the high-coverage genome data for Denisovan and Neanderthal hominids.)

  XLSX, 52K
